# Depression Among the Caregivers of Breast Cancer Patients and its Association with the Quality of Life

**DOI:** 10.2174/17450179-v18-e2208221

**Published:** 2022-10-10

**Authors:** Suzie Y. Rababa’h, Karem H. Alzoubi, Laiali Alquraan, Reema Karasneh, Sayer I Al-azzam, Nasr Alrabadi

**Affiliations:** 1 Department of Medical Science, Irbid Faculty, Al-Balqa Applied University (BAU), Irbid, Jordan; 2 Department of Pharmacy Practice and Pharmacotherapeutics, University of Sharjah, Sharjah, UAE; 3 Department of Clinical Pharmacy, Jordan University of Science and Technology, Irbid, Jordan; 4 Department of Biology, Yarmouk University, Irbid, Jordan; 5 Department of Basic Medical Sciences, Yarmouk University, Irbid, Jordan; 6 Department of Pharmacology, Faculty of Medicine, Jordan University of Science and Technology, Irbid, Jordan

**Keywords:** Beck’s depression inventory, Breast cancer caregivers, Caregiver quality of life, Depression, SF-36, Patients

## Abstract

**Introduction:**

This study investigated the prevalence of depression among the Jordanian caregivers of patients with breast cancer and its effect on their health-related quality of life (QOL).

**Methods:**

This was a cross-sectional study with a sample that consisted of 122 caregivers recruited from 2 hospitals in Jordan over 5 months. A validated questionnaire was used to assess the prevalence of depression symptoms and the aspects of QOL among the participants using Beck’s Depression Inventory-II score and the 36-Item Survey Form (SF-36) score.

**Results and Discussion:**

Depression symptoms were revealed in 27.9% of caregivers. Regarding the QOL, the mental health (MH) subscale was considerably associated with caregivers’ age (P=0.007). The marital status of caregivers was significantly associated with pain (Bodily Pain BP) (*P*=0.015), Beck’s Depression Inventory (BDI; *P*=0.009), and social functioning (SF) (*P*=0.008). The number of caregivers’ siblings was considerably associated with MH (*P*=0.040) subscale. The monthly income of caregivers was associated with BP (*P*=0.042). The residency of caregivers was considerably connected with role limitations because of emotional problems (RE) (P=0.027) and role limitations due to physical health (RF) (P=0.013) subscales. There was a significant correlation between the existing family history of depression with RF (*P*=0.009), RE (*P*=0.005), SF (*P*=0.003), and energy/fatigue (Vitality VT) (*P*=0.001) subscales. Furthermore, the physical activity of caregivers was connected with the RF (*P*=0.030), general health (GH) (*P*=0.018), RE (*P*=0.015), and MH (*P*=0.003) subscales.

**Conclusion:**

Around a third of the caregivers revealed depression symptoms. The QOL subscales for these caregivers were connected with various health and social factors, such as age, number of siblings, marital status, monthly income, residency, family history of depression, and physical activity. The evaluation of the mental and physical well-being of caregivers should always be considered and managed to help them to cope with their QOL.

## 1 INTRODUCTION

Breast cancer is a worldwide public health burden [1, 2], and the second in death cases among Jordanians after cardiovascular diseases [3]. The role of the caregivers is very demanding and affects their mental and physical well-being [4]. Case-control studies revealed the devastating effect of work stress on mental health, especially depression, among caregivers [5, 6]. Caregiving for the vulnerable elderly has been described as a stressful phase that can lead to a lack of mental and physical health of caregivers [7]. Several studies demonstrated a variety of stressors that caregiver health and QOL are affected by, including social, mental functioning, or physical health [8, 9] and behaviors such as reduced exercise or rest and ignoring themselves while working with those patients with breast cancer [9].

Some other studies also revealed that the rate of depression among caregivers of cancer patients were greater than that of the general population [6, 8, 9]. Moreover, they reported that cancer care can lead to an increase in the risk of sleep disturbances, anxiety, depression, and end with decrements in QOL [6, 10]. A cross-sectional study of psychological distress among cancer patients and their family caregivers revealed that both members developed the same levels of distress [11]. This study aims to estimate the prevalence of depression among caregivers and its impact on the mental and healthy functioning of the caregivers of breast cancer patients in Jordan.

## 2 MATERIALS AND METHODS

This study was a cross-sectional where a survey was distributed to the Jordanian caregivers of breast cancer patients between May 2019 and September 2019 at King Abdullah University Hospital (KAUH) and King Hussein Medical Center, which are tertiary care facilities in Jordan hosting more than 400 beds each. The sample of the study included a total of 122 (54 females and 68 male) caregivers of breast cancer patients. The study was approved by the institutional review board at the Jordan University of Science and Technology, Irbid-Jordan (Approval number 66/2018). Informed consent has been obtained from the study participants and Helsinki Declaration has been followed for the study. The participants were interviewed after being introduced to the aim of the study and accepted to provide signed informed consent. The study questionnaires included one for sociodemographic information, another, which was the 36 QOL Short Form (SF-36) Scale, and the third one, which was based on Beck’s Depression Inventory (BDI), concerned with evaluating depression status.

### 2.1 The Sociodemographic Questionnaire

This questionnaire consisted of several questions concerning the caregiver participants' sociodemographic characteristics. It included demographic data, socioeconomic status, the presence of insurance for caregiver cancer patients, and participants’ place of living. It was administered to participants through face-to-face interviews.

### 2.2 The 36 QOL Short Form (SF-36) Scale

The self-rating SF-36 QOL scale included 36 questions measuring the eight aspects or dimensions of the QOL. Each subscale or aspect is measured separately with scores starting from 0 (representing poor and deteriorated QOL) and reaching 100 (representing the optimum QOL). It is worth mentioning that a total score cannot be calculated and each sub-score should be measured separately [12].

### 2.3 Beck Depression Inventory (BDI)

BDI is similarly, a rating scale, used for both apparently healthy and psychiatric patients. This scale can be employed to identify depression among the participants and to measure the change in its severity. Each question on the 21 questions scale was given a weight between 0 and 3 points; therefore, the total score is expected to range between 0 and 63. However, the threshold of the scale cut-off point was set to 17 in the previous validity and reliability Turkish study [13].

### 2.4 Statistical Analysis

The SPSS 16.0 statistics package program (SPSS Inc., Chicago, IL, USA) was used to analyze the collected data. Numerical variables were expressed as means ± standard deviation and categorical variables were expressed as frequencies. The difference between variables was considered significant when the calculated *P-value* was < 0.05. ANOVA test and Student’s *t*-test were used for the numerical variables to compare between groups, while Chi-square test and Pearson’s correlation analysis were used when analyzing the data on categorical variables. The correlation coefficient (*r*) was considered weak when it was from 0.000 to 0.249; moderate from 0.250 to 0.499; and strong from 0.500 to 0.749. While a very strong relationship was considered when it was between 0.750 and 1.000.

## 3 RESULTS

The number of breast cancer caregivers who participated in this study was 122 (n=54 females and 68 males. Almost two-thirds of the caregivers’ participants were married (59.8%), without children, and employed (63.1%). Moreover, a high proportion of caregivers finished high school education (46.7%), while merely 3.3% of caregivers were illiterate. Table **1** summarizes the demographic information of the caregivers' participants. The predominance of depression among patients was 27.9%. As shown in Table **2**, it is distributed as 21.3% with mild depression, 13.1% with moderate depression, and 8.2% with severe depression.

The reliability, variability of scales, and central tendency of the SF-36 QOL survey among the caregivers were shown in Table **3**. Whereas the association between the study variables and the SF-36 QOL subscales was shown in Table **4**. An association between the age of the patients and the MH scale was found (P=0.007). A link was found between the marital status of caregivers and the BP (P=0.015), the BDI (P=0.009), and the SF (P=0.008). As well, the siblings’ number of those caregivers was considerably connected with the mental health (P=0.040) subscale. Moreover, the monthly income of caregivers was associated with BP (P=0.042).

The living place or residency of caregivers was noticeably connected with the RF (P=0.013) and RE (P=0.027) subscales. Caregivers’ educational level, profession, and average sleep hours during the day were not associated with any subscale of the SF-36 QOL questionnaire. Regarding the status of depression, having a history of depression among family members was associated with RF(P=0.009), RE (P=0.005), SF (P=0.003), and VT (P=0.001) subscales. Besides, the physical activity of caregivers was connected with the RF (P=0.030), GH (P=0.018), RE (P=0.015), and MH (P=0.003) subscales. As a final point, the BDI-II index displayed a substantial relationship with marital status (P=0.009) as displayed in Table **4**.

## 4 DISCUSSION

The current study investigated the level of depression and its association with QOL among breast cancer caregivers in Jordan. In line with the cut-off points of the BDI-II rating scale, which was employed to reveal the level of depression and its severity among the caregivers of breast cancer patients, 27.9% of the participants showed clear depression symptoms. Those participants were categorized as follows: caregivers with mild symptoms of depression (21.3%), caregivers with moderate symptoms of depression (13.1%), and caregivers with severe symptoms of depression (8.2%). Considerably, the symptoms of depression among the participating caregivers were significantly linked to their marital status.

A previous study has revealed that the prevalence of depression among breast cancer caregivers was 24.8%)mild, moderate, and severe) in Iran region [14]. Another study, using a validated instrument, showed that approximately 42% of the caregivers for cancer patients were suffering from depression symptoms. This high level of depression prevalence probably affects the QOL of the caregivers [15, 16]. A systematic review using 19 studies focusing on the needs, intervention, motivation of caregivers, and even the consequences of caregiving [17], revealed that the most important correlations with QOL among caregivers were employment status, income, and the severity of their disease. Moreover, the psychosocial challenges were the most predominant among those caregivers [17-19].

The present study compared patients concerning different age groups with the subscales of the SF-36 scoring system; mental health scores of old caregivers were considerably revealed lower than young caregivers. Studies from the literature have revealed a significant and immediate reduction in mental health status after the caregiver period [5, 20] such as the study from Iran which showed that caregivers of breast cancer women who were also family members reported high effects on their quality of life including high psychological stress directly after diagnosis and the subsequent few months [21]. Results of the present study showed that the caregivers (married, single, or others) had reduced scores in BDI-II and social functioning, while their mean bodily pain scores were considerably higher (P < .015). A new study reported that the most widespread problems of physical nature that were expressed by caregivers involved pain, sleep disturbances, fatigue, loss of appetite, loss of physical strength, loss of physical strength, and weight loss [22].

An Australian study reported the direct physical health impact of caregiving, including back, shoulder, and neck problems, tiredness and exhaustion, weight problems, leg and foot problems, arthritis, and stress-related diseases [23, 24]. Another study by Grbich *et al.* (2001)identified that caregivers generally reported strain off the legs and back, with constant tiredness feeling from lifting their patients.

The present study revealed that a higher number of siblings tended to be connected with emotional well-being. Other studies indicated that positive relationships between caregivers and patients and family members were critical as caregivers’ burden becomes important during the period of patients with end-life stages [25]. In families that have less conflict and more interconnected relationships, there is less burden on caregivers as the whole family can help each other in taking care of the sick or elderly members. In agreement with that, the relationship between married couples can also affect the burden on caregivers, whereas couples with pleasant relationships have shown minimal depression and reported better health, and felt less restrained [25].

Moreover, current findings showed that the monthly income of caregivers has been associated with bodily pain, and this may be one of the factors that affected the working hours of the caregivers and their income. This was consistent with other many studies that reported the relationship between the family income and breast cancer caregivers. Two studies informed that caregivers generally lost paid jobs or decreased their working hours as a result of the caregiving role [17, 26, 27]. Other studies explored the factors associated with QOL among caregivers of the same family members in Addis Ababa, Ethiopia [28]. They showed that a low income can affect and reduce the QOL of caregivers [28]. This finding is consistent with other studies where low income has a critical effect on caregivers' QOL and their ability to fulfill their demands, provide proper support for sufficient treatment, and access or use proper health facilities [17, 28]. In another study that was carried out in India, it was shown that caregivers are exposed to depressive disorders [29], despite the patients' stable health and low income. Spousal caregivers, who provide financial support for care and who reside with the patient, as well as the burden of household chores besides caring for patients, are most affected in this study [29].

In the current study, it was found that the residence/current location elucidates significant contributions to the domains of role limitation because of physical health and emotional problems. Current findings are consistent with a study that showed that the health subscale score was higher among caregivers who were living in the same city [30]. Moreover, other previous studies showed that the caregivers who lived in the urban area had lower QOL [30, 31]. When the hospital and place of living are in the same city, this may enable the caregiver and the patient to be familiar with the environment, so they receive more social support, spend less money, and thus the economic burden may be reduced, and they may also feel less anxiety [30, 31]. Furthermore, this study revealed a relationship between family history of depression with RF, RE, SF, and VT subscales. A previous study showed that caregivers who have a history of mental illness are more likely to experience severe distress or emotional disturbance [32]. As a result, caregivers are likely to become more susceptible to depression and stress, and to developing mental illness as the illness continues [32]. Another study showed that depression has become popular among patients with cancer and can influence the disease course, diagnosis of the disease, personal relationships, and the quality and lifestyle of the whole family [33]. The present study also found that the physical activity of caregivers related to the RF, GH, RE, and MH. These results are consistent with previous research which reported that in families of caregivers, the direct impact of physical activity on emotional distress and physical QOL of caregivers has an indirect impact on mental QOL [34]. Furthermore, depression problems are frequent among patients with breast cancer, consequently, having the potential to influence the prognosis of patients' disease, personal relations, clinical course, and the QOL of all family members. Depression effects on life quality can inversely function by reducing the patient’s-QOL, besides increasing the chance of prevention of recovery in patients as well as poorer prognosis in cancer patients [35, 36]. This has been shown in previous studies in several cancers including breast [3], colorectal [37], and hematological [38].

The present study might have some limitations since it included only two hospitals in Jordan. Besides, it was only conducted over 5 months. Thus, it might be limited in terms of the interventional and care services information that was provided to the patients. Further studies are recommended to examine several medical centers and can probably focus on more aspects of patients' services and interventions.

## CONCLUSION

The age, number of siblings, marital status, monthly income, place of residence, family history of depression, and physical activity are all having a detrimental impact on the life quality of cancer caregivers from the same family. Those caregivers may benefit significantly from different kinds of support, including mental, financial, and home care services which in turn can enhance their QOL.

## Figures and Tables

**Table 1:** Demographic Features of Caregivers for Breast Cancer Patients (N=122).

**Demographic Features**	**Frequency**	**Percent%**
**Gender**		
Female	54	44.3
Male	68	55.7
**Age**		
18-30	40	32.8
31-40	38	31.1
41-50	18	14.8
>50	26	21.3
**Nationality**		
Jordanian	120	98.4
Others	2	1.6
**Marital status**		
Married	73	59.8
Single	38	31.1
Others	11	9.0
**Number of siblings**		
None	42	34.4
1-2	26	21.3
3-4	32	26.2
5-6	13	10.7
>6	9	7.4
**Education**		
Illiterate	4	3.3
Primary school	10	8.2
Secondary school	57	46.7
Undergraduate	46	37.7
Graduate studies	5	4.1
Occupation		
**Employed**	77	63.1
Unemployed	17	13.9
Housewife	28	23.0
**Monthly income (JD)**		
<250	30	24.6
250-500	56	45.9
>500	36	29.5
**Living area**		
Urban	78	64.5
Rural	43	35.5
**Average sleep hours/day**		
<4	12	9.9
5-6	51	42.1
7-8	52	43.0
>8	6	5.0
**Family history of depression**		
Yes	19	15.6
No	103	84.4
**Smoking status**		
Smoker	37	30.3
None smoker	85	69.7
**Regular physical activity**		
Yes	24	19.8
No	97	80.2
**Chronic Diseases**		
Diabetes	13	10.8
Hypertension	15	12.5
Cardiovascular diseases	4	3.3
Respiratory diseases	7	5.8
Obesity	6	5
Others	12	10
**Reference person if depressed**		
Family and friends	57	47.9
Health care provider	11	9.2
Social advisor	7	5.9
	44	37.0

**Table 2:** Beck’s depression inventory average -categories and subscales (Cronbach Alpha= 0.884).

-	**No.**	**Percent%**
**BDI mean ± SD (range)**	12.90 ±9.48 (0-46)	
**Minimal range (0-13)**	70	57.4
**Mild (14-19)**	26	21.3
**Moderate (20-28)**	16	13.1
**Severe (29-63)**	10	8.2
Depression (17 cut off point) * **No** **Yes**	88 34	72.1 27.9

**Note:** * “Hisli N. Use of the Beck depression ınventory with Turkish üniversity students: Reliability, validity, and factor analysis. Turk J Psychol. 1989; 7:3–13”.

**Table 3:** Scales of SF-36 quality of life questionnaire of breast cancer patients’ caregivers.

-	**Items**	**Alpha**	**Mean ± SD**
**Physical functioning (PF)**	10	0.909	69.67±28.87
**Role limitations because of physical health (RF)**	4	0.862	61.98±40.96
**Role Limitations-emotional problems (RE)**	3	0.888	62.53±44.00
**Energy (Vitality VT)**	4	0.708	57.34±21.33
**Emotional wellbeing**	5	0.803	63.11±23.08
**Social functioning (SF)**	2	0.657	72.31±25.32
**Pain**	2	0.746	75.70±24.75
**General health (GH)**	5	0.375	63.65±16.22
**Health change**	1	----	52.89±26.85

**Table 4:** Sociodemographic characteristics of caregivers of breast cancer patients with the short form 36 and the parameters pf Becks depression inventory (N= 122).

-	**PF**	**RF**	**RE**	**VT**	**MH**	**SF**	**BP**	**GH**	**BDI**
**Gender**									
Male	71.39±28.57	71.30±37.12	73.46±39.59	61.85±22.87	66.44±23.64	73.84±27.18	84.12±19.46	67.41±15.44	12.69±10.46
Female	68.28±29.25	54.48±42.62	53.73±45.67	53.71±19.43	60.42±22.44	71.08±23.86	68.92±26.54	60.67±16.32	13.07±8.69
***P***	NS	**0.024**	**0.014**	**0.036**	NS	NS	**0.001**	**0.022**	NS
**Age**									
18 – 30	61.41±34.12	50.00±39.74	52.14±45.11	53.68±20.15	57.03±22.30	69.23±26.57	71.47±26.27	63.88±15.13	15.25±9.67
31-40	75.13±26.82	72.37±37.57	64.91±43.11	55.26±21.02	60.95±23.74	72.37±26.02	78.75±24.44	61.18±16.25	13.84±10.67
41-50	68.33±27.33	70.83±41.35	59.26±47.90	58.06±23.40	61.56±25.96	76.39±20.96	68.33±28.04	64.17±20.38	10.83±8.73
>50	75.00±21.82	58.65±44.13	76.92±38.61	65.38±21.12	76.46±16.01	74.04±25.96	82.69±18.44	66.54±15.02	9.35±6.49
***P***	NS	NS	NS	NS	**0.007**	NS	NS	NS	NS
**Marital status**									
Married	70.35±27.71	67.04±39.54	68.06±42.03	59.72±21.08	66.78±23.22	75.00±23.46	80.56±21.16	64.66±16.84	11.05±9.40
Single	71.58±30.74	57.90±40.73	55.26±45.37	55.35±21.70	57.79±22.0	69.08±27.68	71.58±25.29	62.50±15.63	14.79±8.95
Others	58.64±30.09	43.18±47.55	51.52±50.25	48.64±20.63	57.45±23.07	65.91±28.55	58.18±35.13	60.91±14.80	18.64±8.99
***P***	NS	NS	NS	NS	NS	**0.008**	**0.015**	NS	**0.009**
**Number of siblings**									
None	67.38±30.91	57.74±39.62	52.38±44.87	54.64±21.54	56.10±21.34	70.83±27.42	68.99±26.49	63.10±15.85	15.07±8.40
1-2	68.65±30.94	69.23±42.02	70.51±44.55	59.23±21.20	65.08±25.56	78.37±22.52	80.67±25.68	63.65±16.94	13.46±10.26
3-4	71.41±25.22	61.72±43.06	68.75±42.28	56.56±22.56	64.63±24.62	71.48±25.64	80.39±18.06	63.28±16.29	12.81±10.82
5-6	68.08±30.38	63.46±36.25	56.41±41.69	56.15±16.85	66.46±15.54	72.12±18.51	77.69±24.53	63.08±16.40	7.0±5.45
>6	80.63±26.25	59.38±49.89	75.0±46.29	70.42±21.96	82.0±16.84	64.06±32.35	72.81±32.85	68.33±18.37	10.0±8.85
***P***	NS	NS	NS	NS	**0.040**	NS	NS	NS	NS
**Education**									
Illiterate	40.0±52.20	75.0±25.0	77.78±38.49	58.33±27.54	64.0±22.27	79.17±36.08	81.67±31.75	67.50±27.54	6.0±3.92
Primary school	68.0±28.01	55.0±45.34	53.33±50.18	55.0±12.47	52.40±23.88	53.75±28.29	71.0±22.86	57.50±12.08	11.0±8.51
Secondary school	68.25±29.02	67.54±39.52	64.33±43.58	54.27±20.73	60.42±23.59	75.22±22.84	72.76±27.29	62.19±16.28	14.35±9.29
Undergraduate	74.46±27.71	53.80±43.45	60.87±45.16	61.52±23.33	67.65±22.18	72.55±26.56	78.53±22.12	65.54±15.99	12.65±10.14
Graduate studies	63.0±20.80	80.0±20.92	66.67±40.82	58.0±20.80	72.80±19.47	70.0±22.71	89.0±15.47	72.0±14.40	8.0±7.75
***P***	NS	NS	NS	NS	NS	NS	NS	NS	NS
**Occupation**									
Employed	72.21±26.41	64.29±40.84	69.70±41.25	58.96±22.37	65.14±22.72	72.56±25.25	77.63±24.50	64.74±16.12	12.65±9.83
Unemployed	74.71±30.02	66.18±42.33	52.94±47.23	55.20±21.47	62.59±22.22	73.53±27.20	72.50±28.93	62.35±14.37	14.18±8.55
Housewife	59.26±33.24	52.78±40.63	48.15±46.53	54.07±18.24	57.63±24.54	70.83±25.24	72.22±22.94	61.42±17.78	12.82±9.29
***P***	NS	NS	NS	NS	NS	NS	NS	NS	NS
**Monthly income (JD)**									
<250	61.83±29.93	63.33± 40.33	61.11±46.39	56.78±20.62	60.27±22.50	77.50±22.60	67.75±29.45	61.50±15.09	14.73±8.23
250-500	68.73±29.52	59.09±41.21	58.18±44.55	55.27±20.98	60.95±23.66	68.86±26.45	75.23±24.04	62.68±15.98	13.02±10.09
>500	77.64±25.54	65.28±41.95	70.37±41.23	60.97±22.55	68.78±22.26	73.26±25.56	83.06±19.48	66.94±17.41	11.19±9.41
***P***	NS	NS	NS	NS	NS	NS	**0.042**	NS	NS
**Current location**									
Urban	69.04± 28.46	55.13±43.09	55.9829±45.74	57.44±22.56	61.33±25.30	69.87±25.28	74.13±25.44	62.76±17.22	13.94±10.10
Rural	70.81±29.92	74.42±33.85	74.42±38.38	57.17±19.16	66.33±18.21	76.74±25.09	78.55±23.47	64.44±13.46	11.16±8.12
***P***	NS	**0.013**	**0.027**	NS	NS	NS	NS	NS	NS
**Average sleep hours/day**									
<4	62.50±29.81	54.17±45.02	38.89±48.89	50.00±20.11	51.67±21.74	77.08±24.33	59.58±32.91	55.41±17.90	17.50±7.22
5-6	65.60±32.15	58.00±41.17	63.33±42.19	58.70±24.59	63.68±26.23	70.25±25.11	77.00±22.71	64.80±16.70	13.69±10.90
7-8	73.17±25.88	66.35±40.80	66.03±44.52	57.60±16.99	64.08±20.09	73.32±25.96	76.83±24.83	63.65±15.34	10.67±7.27
>8	84.17±18.55	66.67±37.64	66.67±42.16	59.17±31.69	72.67±21.08	70.83±30.28	85.47±13.36	70.83±15.30	13.50±12.87
***P***	NS	NS	NS	NS	NS	NS	NS	NS	NS
**Family history of depression**									
Yes	67.89±26.79	39.47±43.55	36.84±42.88	42.02±19.76	44.63±22.76	56.58±23.71	57.12±27.65	50.00±13.94	21.21±11.57
No	70.00±29.35	66.18±39.28	67.32±42.73	60.17±20.46	66.55±21.55	75.25±24.62	79.17±22.67	66.17±15.39	11.37±8.23
***P***	NS	**0.009**	**0.005**	**0.001**	0.000	**0.003**	0.000	0.000	0.000
**Smoking status**									
Smoker	70.95±24.86	57.43±42.84	59.46±43.84	53.92±21.45	57.84±25.93	70.61±25.55	78.31±24.35	60.41±24.35	15.00±16.93
None smoker	69.11±30.60	63.99±40.21	63.89±44.27	58.85±21.24	65.43±21.46	73.07±25.34	74.56±24.98	65.06±15.80	11.99±8.12
***P***	NS	NS	NS	NS	NS	NS	NS	NS	NS
**Physical activity**									
Yes	71.25±29.86	78.13±31.55	81.94±31.05	63.33±18.75	75.50±20.27	78.13±23.67	79.38±22.68	70.21±12.29	10.29±6.87
No	69.28±28.77	57.99±42.17	57.73±45.53	55.86±21.76	60.04±22.79	70.88±25.63	74. 80±25.26	61.65±16.34	13.61±9.97
***P***	NS	**0.030**	**0.015**	NS	**0.003**	NS	NS	**0.018**	NS
**Chronic diseases**									
**Diabetes**									
Yes	70.0±25.74	48.08±41.41	69.23±39.58	50.38±19.84	59.08±23.73	72.12±21.74	72.88±20.71	48.08±17.39	16.62±12.31
No	69.72±29.21	62.97±40.86	61.01±44.71	57.77±21.42	62.98±22.84	71.82±25.77	75.59±25.27	65.42±15.25	12.62±9.05
***P***	NS	NS	NS	NS	NS	NS	NS	0.000	NS
**Hypertension**									
Yes	73.67±17.88	65.0±39.87	66.67±39.84	58.0±16.23	61.33±26.35	71.67±20.30	70.33±22.75	56.33±21.91	13.07±8.06
No	69.18±30.02	60.82±41.33	61.22±44.82	56.81±22.0	62.73±22.46	71.88±26. 0	76.01±25.05	64.57±15.24	13.05±9.69
***P***	NS	NS	NS	NS	NS	NS	NS	NS	NS
**Cardiovascular diseases**									
Yes	71.25±27.80	75.0±50.0	75.0±50.0	52.08±10.31	68.0±10.33	71.88±15.73	61.88±33.69	50.0±7.07	15.0±11.52
No	69.70±28.90	60.87±40.84	61.45±44.07	57.13±21.58	62.37±23.18	71.85±25.59	75.76±24.45	64.01±16.38	12.98±9.45
***P***	NS	NS	NS	NS	NS	NS	NS	NS	NS
**Respiratory diseases**									
Yes	70.71±25.89	64.29±34.93	61.90±48.80	43.33±15.81	66.86±14.92	67.86±18.90	65.71±29.07	59.29±7.32	16.29±7.11
No	69.69±29.03	61.16±41.49	61.90±44.04	57.81±21.36	62.29±23.30	72.10±25.67	75.90±24.48	63.81±16.72	12.85±9.58
***P***	NS	NS	NS	NS	NS	NS	NS	NS	NS
Obesity									
Yes	78.33±12.11	75.0±31.62	66.67±42.16	44.17±7.36	58.67±16.72	83.33±15.14	70.0±18.91	54.17±19.60	11.83±5.15
No	69.29±29.33	60.62±41.43	61.65±44.37	57.64±21.59	62.76±23.19	71.24±25.60	75.58±25.06	64.03±16.10	13.11±9.66
***P***	NS	NS	NS	NS	NS	NS	NS	NS	NS
**Reference person if feeling depressed**									
Family and friends	70.80±26.54	63.84±39.86	67.86±42.15	58.84±19.75	62.93±19.85	73.21±23.65	74.46±26.37	63.42±15.79	12.44±9.21
Health care provider	72.73±26.68	47.73±50.56	60.61±49.03	58.18±23.16	63.27±23.85	63.64±24.66	88.18±18.20	58.64±20.01	15.09±10.15
Social advisor	71.43±39.76	78.57±36.60	76.19±41.79	62.62±14.27	80.57±8.77	85.71±15.19	76.07±30.34	76.43±17.01	13.29±10.10
No one	66.48±31.38	58.52±41.04	53.03±45.65	53.18±23.73	58.55±26.63	69.89±28.57	73.18±23.52	62.16±14.92	13.41±9.76
***P***	NS	NS	NS	NS	NS	NS	NS	NS	NS

**Note:** The study used One-way ANOVA to compare variables with three or more categories, whereas it used unpaired t-test for two-group variable.**Abbreviations:** RP, role-physical; RE, role-emotional; PF, physical functioning; VT, vitality; MH, mental health; BP, bodily pain; SF, social functioning; BDI, Beck’s Depression Inventory; GH, general health; JD, Jordanian Dinar (=0.71 US Dollars).

## Data Availability

Data will be available upon request via e-mailing the corresponding author [S.Y.R].

## LIST OF ABBREVIATIONS

QOL: = Quality of Life
MH: = Mental Health
